# Neural evidence for procedural automatization during cognitive development: Intraparietal response to changes in very-small addition problem-size increases with age

**DOI:** 10.1016/j.dcn.2023.101310

**Published:** 2023-10-04

**Authors:** Andrea Díaz-Barriga Yáñez, Léa Longo, Hanna Chesnokova, Céline Poletti, Catherine Thevenot, Jérôme Prado

**Affiliations:** a Centre de Recherche en Neurosciences de Lyon (CRNL), INSERM U1028 - CNRS UMR5292, Université de Lyon, France; b Institut de Psychologie, Université de Lausanne, Switzerland

**Keywords:** Development, Arithmetic, Procedure, Problem-size effect, FMRI

## Abstract

Cognitive development is often thought to depend on qualitative changes in problem-solving strategies, with early developing algorithmic procedures (e.g., counting when adding numbers) considered being replaced by retrieval of associations (e.g., between operands and answers of addition problems) in adults. However, algorithmic procedures might also become automatized with practice. In a large cross-sectional fMRI study from age 8 to adulthood (n = 128), we evaluate this hypothesis by measuring neural changes associated with age-related reductions in a behavioral hallmark of mental addition, the *problem-size effect* (an increase in solving time as problem sum increases). We found that age-related decreases in problem-size effect were paralleled by age-related increases of activity in a region of the intraparietal sulcus that already supported the problem-size effect in 8- to 9-year-olds, at an age the effect is at least partly due to explicit counting. This developmental effect, which was also observed in the basal ganglia and prefrontal cortex, was restricted to problems with operands ≤ 4. These findings are consistent with a model positing that very-small arithmetic problems–and not larger problems–might rely on an automatization of counting procedures rather than a shift towards retrieval, and suggest a neural automatization of procedural knowledge during cognitive development.

## Highlights

• Does cognitive development involve procedural automatization?
• We tracked correlates of the arithmetic problem-size effect from age 8 to adulthood.
• Young children showed intraparietal activity partly associated with counting.
• We found age-related increases of activity in that region as performance improved.
• This effect was specific to very-small problems, supporting procedural automatization.

## 1 Introduction

Children’s cognitive development is often thought to involve qualitative changes in the distribution of problem-solving strategies, with a shift from inefficient procedures to efficient memory-based strategies (Siegler, 2016, Siegler, 1999). Although this model has been applied to a range of domains, including reading, number processing, social cognition and tool use (Amsterlaw and Wellman, 2006, Davis and Evans, 2021, Forestier and Oudeyer, 2016, Opfer and Siegler, 2007, Rittle‐Johnson and Siegler, 1999), a classic illustration comes from mental arithmetic (Geary et al., 2012). For example, seminal studies have shown that children start solving small additive problems (e.g., 2 + 3 = 5) by using explicit counting, a procedure that is cost-intensive and still clearly used by at least half of them up to the age of 9 (Ashcraft and Fierman, 1982, Siegler, 1987, Thevenot et al., 2016). After the age of 10, however, most children report retrieving answers of these problems without counting silently (Geary et al., 2012). The classic explanation for this effect is that after the age of 10 answers would have been computed so many times that operands and answers should be associated in long-term memory (Ashcraft, 1987, Campbell and Oliphant, 1992, Chen and Campbell, 2018, Logan, 1988, Siegler and Shrager, 1984).

**Fig. 1:**
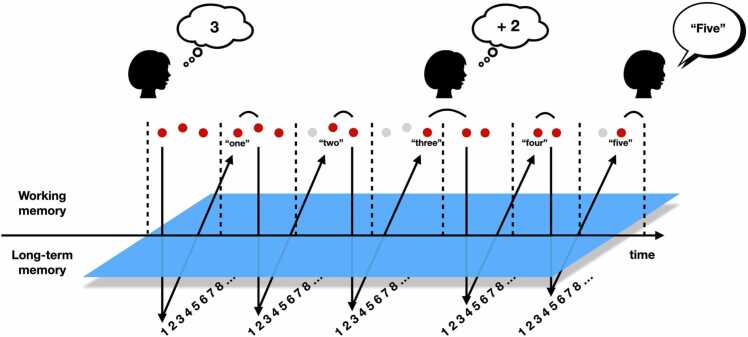
Automatized counting would consist in a rapid scan of a representation of each operand, recursively accessing the number sequence stored in long-term memory and incrementing each item with the next numerical value from the sequence. The figure depicts how the addend ‘2′ would be combined to the augend ‘3′ to produce the answer ‘5′.

Nevertheless, algorithmic procedures that are initially cost-intensive may also become compiled and automatized through practice (Anderson et al., 2004, Logan and Crump, 2011). In other words, cognitive development may also be characterized by *quantitative* changes in problem-solving strategies. When adding single-digit numbers, for example, silent counting might be practiced so frequently by children that it might progressively turn into fast mental scanning of a sequence of numbers, a process that has been termed *automatized counting* (Barrouillet and Thevenot, 2013, Fayol and Thevenot, 2012, Thevenot et al., 2016, Uittenhove et al., 2016). This developmental change would be associated with a significant increase in procedural efficiency. Indeed, because the cost of counting is estimated at around 125 ms per item (Landauer, 1962), silent counting is accompanied by a relatively large increase in solution times as problem sum increases, a phenomenon called the problem size effect (PSE) (Zbrodoff and Logan, 2005). But scanning a mental representation might be done at a much faster rate. For instance, studies using memory span tasks indicate that scanning a list of words for immediate serial recall may only take tens of ms per item (Cowan et al., 2002, Sternberg, 1969). As argued previously (Barrouillet and Thevenot, 2013, Uittenhove et al., 2016), this scanning rate may be achieved in mental arithmetic only when magnitudes of operands can be represented within a single focus of attention, i.e., when operands are ≤ 4 (so that no more than four successive numbers would be accessed at once) (Uittenhove et al., 2016). Thus, the automatized counting model (see Fig. 1) posits that adding operands ≤ 4 could specifically involve a fast mental scanning of a numerical sequence in adults, potentially leading to a still reliable but much smaller PSE. Adding operands ≥ 5 might be associated with a mixture of more costly procedures (e.g., silent counting) or direct retrieval from memory. In essence, this distinction between problems with operands ≤ 4 and operands ≥ 5 echoes the distinction between subitizing and counting in enumeration tasks (the former being associated with a cost by item much smaller than the latter, Mandler and Shebo, 1982).

Automatized counting model, adapted from Uittenhove et al. (2016).

It is difficult to distinguish automatized counting from retrieval at the behavioral level and there is much debate regarding the plausibility of the account (Chen and Campbell, 2018, Thevenot and Barrouillet, 2020). For instance, studies have found a small (i.e., down to 20–40 ms) but reliable PSE for addition problems with operands ≤ 4 in adults and adolescents, whereas no PSE was found for small problems (up to a sum of 10) with operands ≥ 5 (Bagnoud et al., 2021, Poletti et al., 2023, Uittenhove et al., 2016). Some have claimed that this pattern provides evidence for a specific use of automatized counting when operands are ≤ 4 (Uittenhove et al., 2016). Others, however, have argued that the small PSE for problems with operands ≤ 4 may stem from memory interferences, which typically increase with the problem sum (e.g., larger problems share a sum with other problems more often than smaller problems do) (Chen and Campbell, 2018). It has also been argued that the lack of PSE for problems with operands ≥ 5 may be more apparent than real, as it may be driven by the particular status of sum-to-10 problems which are easier to solve and artificially decrease the PSE (Chen and Campbell, 2018). When these problems are removed, a PSE may appear even for problems with operands ≥ 5 (which may again be due to interference from memory rather than automatized counting; Chen and Campbell, 2018). Therefore, the doubt remains regarding the existence of automatized counting.

However, the retrieval and the automatized counting accounts make different hypotheses at the neural level. On the one hand, a counting to retrieval shift in strategy would suggest qualitative changes in neural mechanisms supporting the PSE with development. For example, the brain regions in which activity is associated with changes in problem-size in 8- and 9-year-olds (who still rely on counting for half of them; Ashcraft and Fierman, 1982) may no longer respond to changes in problem-size in adults and children older than 10 (who are assumed to all retrieve results from memory), such that other regions should support the PSE after that age. On the other hand, the automatized counting view argues that a similar procedure is automatized and used throughout development. Therefore, the brain regions in which activity is associated with changes in problem-size in 8- and 9-year-olds should still respond to changes in problem-size in adults and children older than 10. In fact, these regions might even become *increasingly* sensitive to changes in problem size as the procedure becomes increasingly efficient with age. However, automatized counting should be restricted to problems with operands ≤ 4. Therefore, the predictions above would only apply to problems involving operands ≤ 4. To our knowledge, the automatized counting view is the only theory that predicts a neural dissociation between the neural substrates of the PSE for problems with operands ≤ 4 and those with operands ≥ 5.

**Fig. 2:**
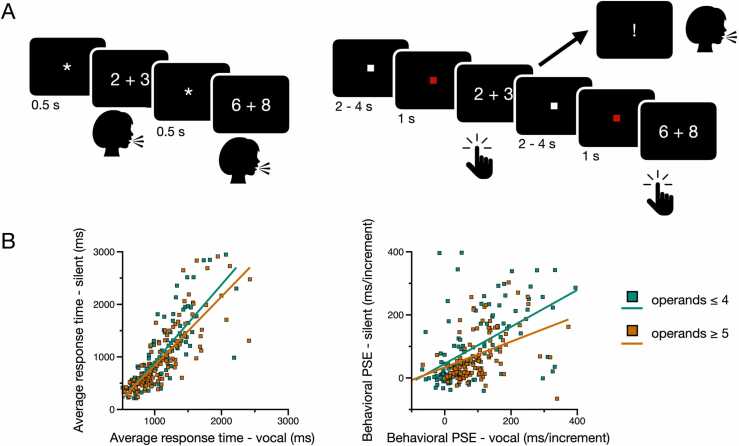
Vocal and silent arithmetic tasks. (A) Outside of the scanner (left), participants vocally produced the answer of single-digit addition problems. Response times were measured using a vocal key. In the scanner (right), participants mentally produced the answer of single-digit addition problems. They were instructed to press on a button when the answer was reached. In some trials, they were cued to produce the answer vocally. (B) Scatter plots showing the relation between average response times (left) and slopes of the PSE (right) measured from the vocal and silent arithmetic tasks.

The present study aimed to assess the above hypotheses. Specifically, we used functional magnetic resonance imaging (fMRI) to track the changes in brain activity associated with the PSE while 8–9-year-olds (n = 31), 11–12-year-olds (n = 31), 14–15-year-olds (n = 26), and adults (n = 40) were asked to mentally produce the result of small addition problems (problems with sums ≤ 10) in the scanner (see Fig. 2**A**). Ten is believed to be a pivotal age from which most participants are believed to retrieve answer from memory according to memory retrieval accounts (Ashcraft and Fierman, 1982). Therefore, brain regions in which activity increased with problem size (i.e., regions showing a *neural* PSE) in 8–9-year-olds served as regions of interest (ROIs) in the analyses of the three other groups to evaluate (i) whether and to what extent these ROIs are sensitive to the PSE in adults and children older than 10 and (ii) whether the relation between neural activity and problem size (the neural PSE) depends on whether the magnitude of the operands is within a single focus of attention (operands ≤ 4) or not (operands ≥ 5).

## 2 Method

### 2.1 Participants

One hundred and forty-nine children, adolescents, and adults between the ages of 8 and 30 were recruited for the study. Participants were contacted through advertisements on social media. They were divided into four age groups: 8- to 9-year-olds (hereafter 8–9-yo, n = 41), 11- to 12-year-olds (hereafter 11–12-yo, n = 36), 14- to 15-year-olds (hereafter 14–15-yo, n = 30), and adults over the age of 18 (n = 42). The study consisted in two separate testing sessions: one behavioral session (which included psychometric and behavioral testing, as well as mock MRI scanning for children and adolescents) and one fMRI session. Seven participants were excluded from the final analyses because they did not complete the fMRI session (8–9-yo, n = 3; 11–12-yo, n = 2; 14–15-yo, n = 2). Fourteen participants were also excluded after fMRI preprocessing, either because of excessive head motion in the scanner (see criteria below) or poor image quality (8–9-yo, n = 7; 11–12-yo, n = 3; 14–15-yo, n = 2; adults, n = 2). The final sample (n = 128) consisted in 31 participants in the 8–9-yo group, 31 in the 11–12-yo group, 26 in the 14–15-yo group, and 40 adults (see Table 1 for demographic information). All participants were native French speakers. Participants below the age of 18 gave their assent to participate in the study, while their parents gave written informed consent. Adult participants gave written informed consent. The study was approved by a French ethics committee (Comité de Protection des Personnes Est 4, IDRCB 2019-A01918–49). Participants (or parents if participants were below 18) were paid 40 euros per session for their participation.

**Table 1:** Demographic information and average behavioral measures.

Measure	8–9-yo (n = 31) Mean (SD)	11–12-yo (n = 31) Mean (SD)	14–15-yo (n = 26) Mean (SD)	Adults (n = 40) Mean (SD)
*Demographic information*				
Age (in years)	8.95 (0.62)	11.96 (0.63)	14.72 (0.52)	22.57 (2.41)
Male/Female	16/15	18/13	13/13	15/25
*IQ (WISC / WASI)*				
Similarities score^1^	113 (10)	112 (10)	105 (14)	113 (13)
Raven’s matrices score^1^	106 (12)	104 (13)	99 (11)	93 (14)
*Reading fluency (Alouette*)				
Efficiency score^2^	270 (91)	396 (83)	450 (90)	556 (102)
*Arithmetic fluency (WJ-III)*				
Number correct answers^2^	50 (14)	74 (17)	88 (17)	109 (20)
*Processing speed*				
Choice reaction time^3^	531 (108)	414 (46)	362 (40)	343 (43)
*Vocal arithmetic task*				
Percent correct^4^	98 (3)	98 (2)	99 (1)	99 (2)
Response time (operands ≤ 4)^4^	1364 (586)	1084 (432)	949 (348)	796 (263)
Response time (operands ≥ 5)^4^	1463 (668)	1100 (427)	963 (348)	804 (276)
*Silent arithmetic task*				
Percent correct^4^	95 (8)	92 (10)	97 (7)	97 (3)
Response time (operands ≤ 4)^(4)^	1680 (1055)	958 (637)	724 (425)	562 (303)
Response time (operands ≥ 5)^(4)^	1614 (988)	924 (559)	722 (419)	571 (329)

**Notes.**

^1^standardized score (Mean = 100, SD = 15),

^2^raw score (see Methods)

^3^all trials (in millisecond),

^4^non-tie problems only (in millisecond).

### 2.2 Psychometric testing

Most participants were tested on measures of IQ (n = 127), reading fluency (n = 127), arithmetic fluency (n = 125), and processing speed (n = 125) during a behavioral session. First, Intelligence Quotient (IQ) was measured using the similarities and the matrix reasoning subtests of either the Wechsler Intelligence Scale for Children - Fifth Edition (WISC-V) (Wechsler, 2014) for children and adolescents or the Weschler Adult Intelligence Scale 4th Edition (WAIS-IV) for adults (Wechsler, 2008). The similarities subtest, which measures verbal comprehension, requires participants to find similarities between pairs of words. The matrix reasoning subtest, which measures nonverbal reasoning, requires participants to find a picture missing from a matrix grid of other abstract pictures. Second, reading fluency was assessed using the Alouette-R test (Lefavrais, 2005). Participants have to read out loud a 265-word nonsensical text as quickly and as accurately as possible in a maximum of 3 min. Based on accuracy and reading time, we computed an efficiency score (i.e., CTL), using the following formula (Pourcin et al., 2016): CTL = (A/RT)* 180, where A = accuracy (self-corrections included), and RT = reading time (maximum = 180 s). Third, arithmetic fluency was assessed using the Math Fluency subtest of the Woodcock-Johnson III (WJ-III) (Woodcock et al., 2001). In this subtest, participants have to solve as many single-digit addition, subtraction, multiplication, and division problems as they can within 3 min. The number of correctly solved problems is measured. Finally, processing speed was assessed using a computer-based choice reaction time task, controlled by the DmDX software (Forster and Forster, 2003). The task required participants to press as quickly and accurately as possible on either a left or a right key every time an arrow pointing to either the left or the right was shown on the screen (40 trials). Each arrow was presented for up to 10 s or disappeared as soon as a response was recorded. Correct reaction times were measured from the onset of the stimulus presentation to the onset of the response.

### 2.3 Familiarization with the fMRI environment

On the day of psychometric testing, children and adolescents were familiarized with the fMRI environment in a mock scanner. They listened to a recording of the noises associated with all fMRI sequences. A motion tracker system (3D Guidance track STAR, Ascension Technology Corporation) was used to measure head movements and provide online feedback to participants. Finally, participants practiced 25 trials of the silent arithmetic task in that mock scanner.

### 2.4 Vocal arithmetic task (out-of-scanner)

During the behavioral session, all participants also performed a version of the vocal arithmetic task used by Uittenhove et al., 2016 (see Fig. 2A, left). In this task, participants are presented with a series of single-digit addition problems and have to say the answer out loud. Stimuli were all 45 single-digit addition problems with a sum less than or equal to 10, in both commutative orders (e.g., 2 + 3 and 3 + 2). Each problem was repeated three times over three different blocks, for a total of 135 trials. Trial order was randomized within each block. The experiment was controlled by the DmDX software (Forster and Forster, 2003). Vocal responses were recorded with a voice key and individually checked off-line for accuracy using CheckVocal (Protopapas, 2007). CheckVocal was also used to manually adjust the latencies recorded by DmDX, if necessary (e.g., when participants coughed or hesitated before giving their answer). Each trial began by the presentation of a 500 ms fixation signal represented by a “* ”, followed by the presentation of the addition problem. The problem was displayed on screen until the onset of the verbal response was detected by the voice key or for 10,000 ms if no response was recorded. Participants were instructed to give their answer as quickly and accurately as possible.

### 2.5 Silent arithmetic task (in-scanner)

In the MRI scanner, participants once again performed a version of the arithmetic task described above, with three main differences (see Fig. 2A, right). First, each single-digit problem was presented five times (rather than three times) over five consecutive runs to maximize signal to noise ratio in the fMRI scanner, for a total of 225 trials. Second, participants were instructed to not say the answer of each problem out loud but rather to press on a button with their right hand as soon as they could come up with the answer in their head. This was meant to minimize head motion while ensuring that the task remained a production and not an evaluation task. However, to ensure that participants followed instructions and had the answer in their mind when they pressed on the response key, some problems were immediately followed by an exclamation point that appeared on the screen for 1000 ms. In such cases, participants had to quickly say the answer out loud before the disappearance of the exclamation point. Answers were recorded manually by the experimenter. There were four arithmetic problems of such kind for every run of 45 trials. Third, the timing of the task was adapted to the constraints of fMRI. Specifically, each problem (which remained on the screen until a response was detected) was preceded by a red square for a fixed duration of 1000 ms and followed by a variable period of fixation (i.e., a white square) from 2000 to 4000 ms.

Trial order within each run and run order was randomized to create four different scenarios, which were presented to participants in a counterbalanced order. Trials in which a vocal response was required were pseudo-randomized in the trial list, such that these trials were not located at the beginning of a run and were not following each other’s. The task was programmed and presented with PsychoPy3 (v2020.2.5). A screen was installed at the end of the scanner bore and visual stimuli were displayed by a projector in the room adjacent to the scanner. A 45° mirror was placed over the headrest so that participants could view the screen by looking upward. Head movement was minimized during the scan by cushions placed around the participant’s head.

### 2.6 Behavioral analysis

In both the vocal and silent arithmetic tasks, trials with RTs more than 6000 ms or less than 100 ms were considered outliers and were excluded from these analyses. Trials in which participants were required to provide a vocal answer in the silent arithmetic task were also excluded from the analyses as they were not included in the fMRI analyses (see below). After examining whether results from the vocal task replicated the patterns of RTs found in our previous studies (Poletti et al., 2023, Uittenhove et al., 2016), we excluded from the main analyses the trials with tie problems (i.e., problems with two operands that were identical) and the trials with problems for which the sum was equal to 10. This is because ties and sum-to-10 problems may have a special status in memory and their RTs may stand out from other single-digit addition problems (Uittenhove et al., 2016). Response times (RTs) associated with the remaining trials were analyzed as a function of both operand magnitude (problems with operands ≤ 4, problems with operands ≥ 5) and problem sum (3, 4, 5, 6, 7 for problems with operands ≤ 4; 6, 7, 8, 9 for problems with operands ≥ 5). For each participant, task, and problem category, we then calculated the slope of the PSE. Slope values were submitted to second-level one-sample t-tests across participants to assess whether the average slope of the PSE was greater than 0. One-tailed p values less than 0.05 were considered to be significant.

### 2.7 fMRI data acquisition

Images were collected using a Siemens Prisma 3 T MRI scanner with a 64-channel receiver head-neck coil (Siemens Healthcare, Erlangen, Germany) at the CERMEP Imagerie du vivant in Lyon, France. The blood oxygenation level-dependent (BOLD) signal was measured with a susceptibility-weighted single-shot echo planar imaging sequence. Imaging parameters were as follows: repetition time (TR) = 2000 ms, echo time (TE) = 24 ms, flip angle = 80°, field of view (FOV) = 220 × 206 mm2, resolution = 1.72 × 1.72 mm2, slice thickness = 3 mm (0.48 mm gap), number of slices = 32. A high-resolution T1-weighted whole-brain anatomical volume was also collected for each participant. Parameters were as follows: TR = 2400 ms, TE = 2.81 ms, flip angle = 8°, FOV = 224 × 256 mm2, resolution = 1.0 × 1.0 mm2, slice thickness = 1.0 mm, number of slices = 192.

### 2.8 fMRI data pre-processing

Images were analyzed using SPM12 (http://www.fil.ion.ucl.ac.uk/spm, Welcome department of Cognitive Neurology, London, UK) in Matlab R2020b. Each fMRI run started with 2 dummy scans to allow for magnetization equilibration effects. Functional images were corrected for slice acquisition delays and spatially realigned to the first image of the first run to correct for head movements. Realigned images were smoothed with a Gaussian filter (4 mm × 4 mm × 7 mm full-width at half maximum). ArtRepair (https://www.nitrc.org/projects/art_repair/) was used to suppress residual fluctuations due to large head motion and identify volumes with significant artifact and outliers relative to the global mean signal. Functional volumes with a global mean intensity greater than 3 standard deviations from the average of the run or a volume-to-volume motion greater than 2 mm were identified as outliers and substituted by the interpolation of the 2 nearest non-repaired volumes. Participants with outliers in more than 20% of volumes were excluded from the analyses. After outlier exclusion, there was no difference between the groups with respect to translational (x, y, z) or rotational (pitch, roll, yaw) movements (all ps > 0.30).

Finally, functional images were normalized into the same stereotaxic space. Studies have found that anatomical differences between children older than 7–8-year-olds and adults are small enough that they are beyond the resolution of fMRI experiments (Kang et al., 2003). Therefore, considering the age of our participants, the resolution of our data, and the fact that we wanted to be able to compare the four groups within the same space, we normalized all individual brains into the standard adult Montreal Neurological Institute (MNI) space. This was done in two steps. First, after coregistration with the functional data, the structural image was segmented into gray matter, white matter, and cerebrospinal fluid by using a unified segmentation algorithm (Ashburner and Friston, 2005). Second, the functional data were normalized to the MNI space by using the normalization parameters estimated during unified segmentation (normalized voxel size, 2 mm^3^ × 2 mm^3^ × 3.5 mm^3^).

### 2.9 fMRI data processing

Event-related regression analyses (conducted separately in each participant) were performed using a version of the general linear model in which the fMRI signal associated with each arithmetic problem was modeled as an epoch that started with the presentation of the problem and ended with the participant’s response. In each run, trials with RTs more than 6000 ms or less than 100 ms were excluded from behavioral and fMRI analyses. Trials in which participants were required to provide a vocal answer were excluded from the analyses to avoid contaminating brain activity with head motion. Trials with tie problems and trials with problems for which the sum was equal to 10 were also excluded from the analyses.

The remaining correct trials were sorted by operand magnitude (problems with operands ≤ 4, problems with operands ≥ 5). For each of these categories, an additional parametric regressor encoding the sum associated with each problem was added in the design matrix. This led to four regressors per run: one regressor coding for the average activity associated with problems with operands ≤ 4, one regressor coding for the average activity associated with problems with operands ≥ 5, one parametric regressor coding for the change in activity as the sum of problems with operands ≤ 4 increased, one parametric regressor coding for the change in activity as the sum of problems with operands ≥ 5 increased. These parametric regressors allowed us to estimate the slope of the PSE within each problem category. Epochs were convolved with a canonical hemodynamic response function (HRF). Time series data from each run were then high-pass filtered (1/128 Hz) and serial correlations were corrected using an autoregressive AR(1) model.

### 2.10 Voxelwise analyses

For each participant, beta values associated with the parametric regressors were submitted to second-level one-sample t-tests across the whole brain, as well as to a one-way ANOVA with age group as a between-subject factor. This allowed us to identify brain regions in which activity increased with problem sum in a given group and whether that effect differed as a function of group. The resulting t-maps were thresholded using the non-parametric permutation-based Threshold-Free Cluster Enhancement (TFCE) method (Smith and Nichols, 2009), implemented in the TFCE Toolbox r164 (http://dbm. neuro.uni-jena.de/tfce/). Based on previous literature (Sokolowski et al., 2022), we anticipated that a region responsive to problem-size might be the intraparietal sulcus (IPS), which is central to numerical cognition (Nieder, 2016). Therefore, clusters were considered significant if they survived a Family-Wise Error (FWE) rate corrected threshold of p < .05 (Bennett et al., 2009), either across the whole-brain or within an anatomical mask of the IPS (i.e., small volume correction) defined using the Anatomy Toolbox v2.2 (Eickhoff et al., 2005). The IPS mask, which was used in several of our previous studies (Girard et al., 2022, Schwartz et al., 2018), consisted in voxels with at least 50% probability of belonging to one of the IPS subdivisions as defined in the Anatomy Toolbox (hIP1, hIP2, and hIP3).

### 2.11 Functional ROIs

Functional ROIs were brain regions that were associated with a significant neural PSE in the 8–9-yo group. Functional ROIs included all voxels within a 6-mm radius of each coordinate of interest. We calculated for each participant the average activity associated with each parametric regressor within an ROI by averaging the beta values across all voxels within that ROI. Note that data from one participant were removed from the analyses because averages differed by more than 2.5 SD from the mean of all participants. Average beta values were then submitted to second-level one-sample t-tests across participants from the 8–9-yo group to identify ROIs for which the slope of the PSE was greater than 0. One-tailed p values less than 0.05, corrected for multiple comparisons across all functional ROIs using the Bonferroni method, were considered to be significant.

### 2.12 Anatomical ROIs

Additional analyses focused on anatomical ROIs of the dorsal striatum, given the central role of this structure in procedural learning and the prediction that counting procedures may become automatized throughout development (Janacsek et al., 2022). Using wfu_pickatlas, we defined left and right ROIs in the caudate nucleus and the putamen. ROIs included all voxels within each mask. As above, we calculated for each participant the average activity associated with each parametric regressor within an ROI by averaging the beta values across all voxels within that ROI. Average beta values were then submitted to second-level one-sample t-tests across participants to identify ROIs for which the slope of the PSE was greater than 0. One-tailed p values less than 0.05, corrected for multiple comparisons across all anatomical ROIs using the Bonferroni method, were considered to be significant.

### 2.13 Data availability

The experimental paradigms, anonymized behavioral data, individual beta values extracted from the ROIs, as well as the ROI images and the whole-brain unthresholded and thresholded t-maps corresponding to Fig. 5 and Fig. 7 are publicly available via OSF at [https://doi.org/10.17605/OSF.IO/7TDFU].

**Fig. 3:**
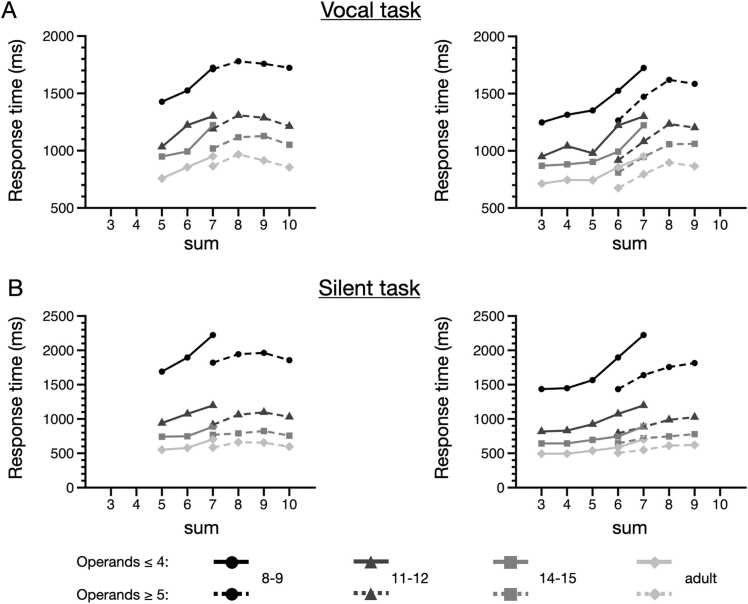
Mean response times for each age group (8–9-yo, 11–12-yo, 14–15-yo, adult) and each problem category (operands ≤ 4 and operands ≥ 5) according to the sum of the problem. (A) In the vocal task, patterns of RTs for sets of problems excluding ties and 1-problems, while including sum-to-10 problems (left) and patterns of RTs for sets of problems excluding ties and sum-to-10 problems, while including 1-problems (right). (B) In the silent task, patterns of RTs for sets of problems excluding ties and 1-problems, while including sum-to-10 problems (left) and patterns of RTs for sets of problems excluding ties and sum-to-10 problems, while including 1-problems (right).

**Fig. 4:**
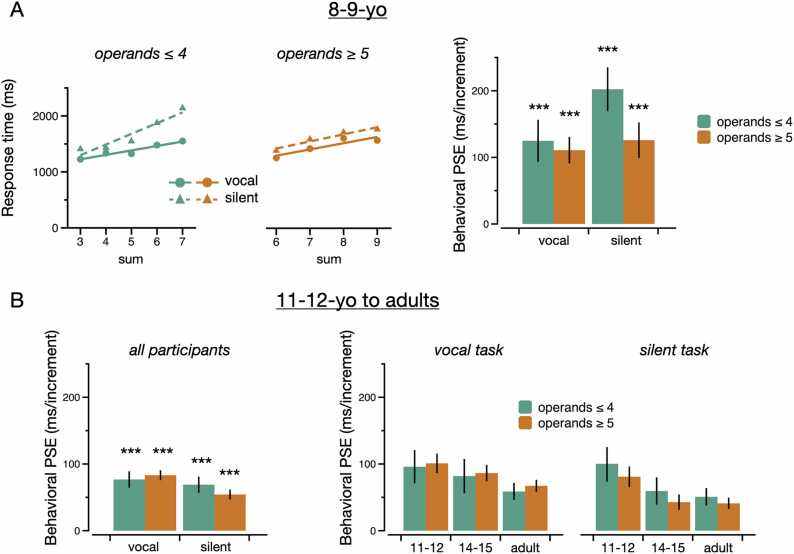
Behavioral PSE. (A) Across participants from the 8–9-yo group, scatter plot showing the relation between RT and problem sum (left) and bar graph showing the size of the PSE (right) as a function of both task (vocal, silent) and magnitude of operands (≤ 4 and ≥ 5). (B) Across participants from the three other groups (11–12-yo to adults), bar graph showing the size of the PSE as a function of task (left) as well as a function of both task and age group (right). Error bars represent standard error of the mean. *** , p < 0.001.

**Fig. 5:**
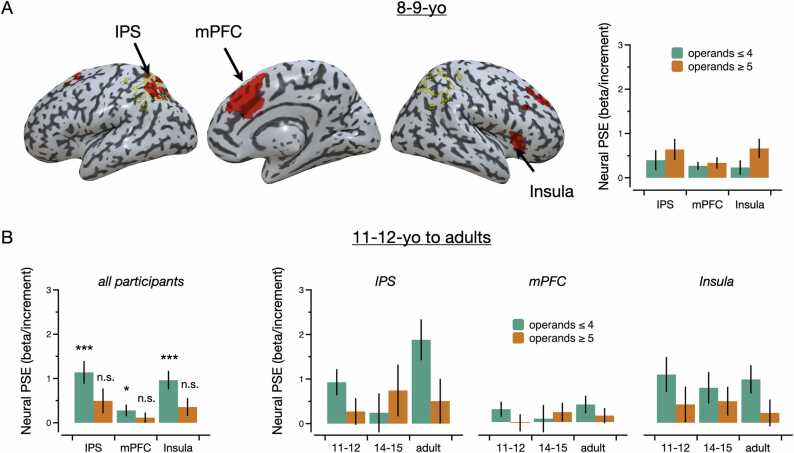
Functional ROI analyses. (A) Across participants from the 8–9-yo group, brain regions showing a positive relation between RT and problem sum (i.e., a neural PSE) overlaid onto an inflated rendering of the brain (left). The yellow outline shows anatomical boundaries of the IPS. The bar graph on the right shows the neural PSE as a function of ROI and magnitude of operands (≤ 4 and ≥ 5). Note that the graph is presented for illustrative purpose only as activity was not extracted from an independent contrast. (B) Across participants from the three other groups (11–12-yo to adults), bar graphs showing the size of the neural PSE as a function of ROI (left) as well as a function of both ROI and age group (right). Error bars represent standard error of the mean.

**Fig. 6:**
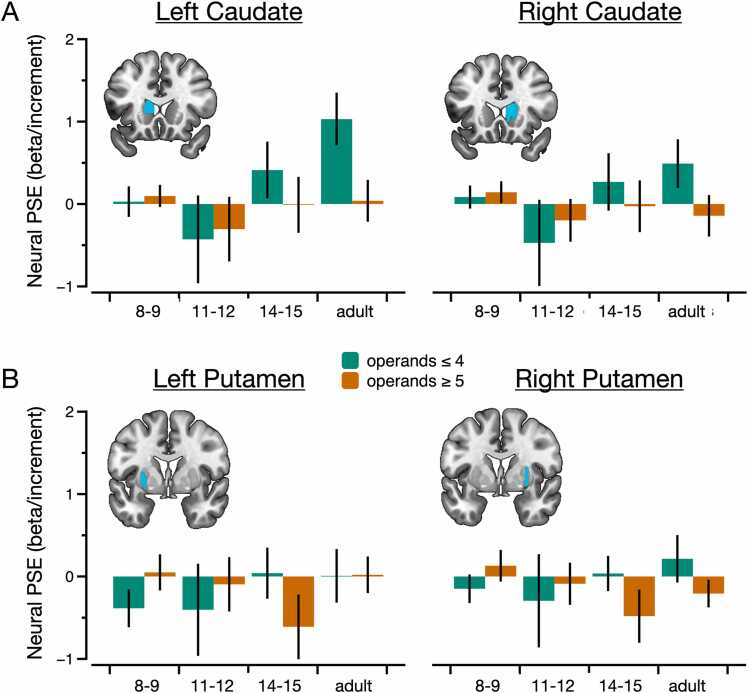
Anatomical ROI analyses. (A) Bar graphs showing the size of the neural PSE in the caudate nucleus as a function of age group and laterality. (B) Bar graphs showing the size of the neural PSE in the putamen as a function of age group and laterality. Error bars represent standard error of the mean.

**Fig. 7:**
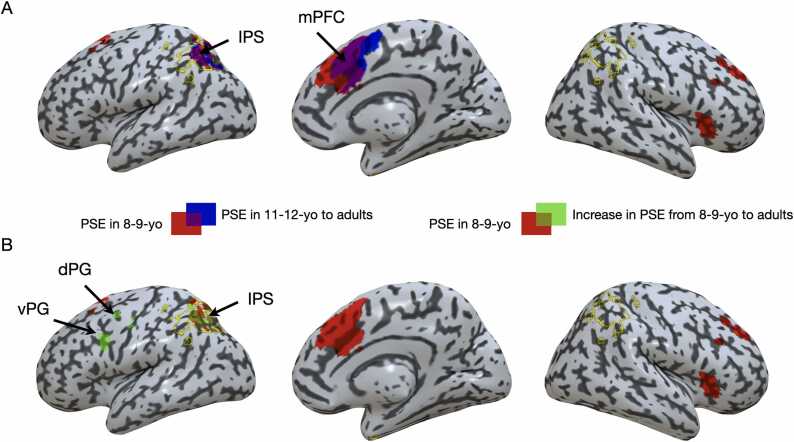
Neural PSE (whole-brain analyses). (A) Brain regions showing a positive relation between RT and problem sum (i.e., a neural PSE) for participants younger (red) and older (blue) than 10 overlaid onto an inflated rendering of the brain. (B) Brain regions showing an increase in neural PSE with age (green) overlaid onto an inflated rendering of the brain that also shows the neural PSE for the 8–9-yo group. The yellow outline shows anatomical boundaries of the IPS. dPG, dorsal precentral gyrus, vPG, the ventral precentral gyrus.

### 2.14 Code availability

The Matlab scripts used to analyze the fMRI data are available in GitHub, [https://github.com/BBL-lab/BBL-batch-system].

## 3 Results

### 3.1 Behavioral results: descriptive statistic and qualitative comparison with previous studies

Descriptive statistics for psychometric measures for the four groups of participants are shown in Table 1. IQ scores, which were obtained from the WISC in children and adolescents and from the WAIS for adults, were within the average to high-average range in each group. Reading and arithmetic fluency scores (as measured with the Alouette and the WJ-III) were also within the normal range in each group (Lefavrais, 2005, Woodcock et al., 2001). As expected, reading fluency (F_3123_ = 56.6, p < 0.001, η²p = 0.58), arithmetic fluency (F_3121_ = 69.7, p < 0.001, η²p = 0.63), and processing speed (F_3121_ = 53.2, p < 0.001, η²p = 0.57) all improved with age.

We first evaluated whether patterns of RTs obtained in the vocal and silent tasks were similar to patterns observed in previous studies, in which there was a positive PSE for small problems with operands ≤ 4 and a lack of PSE for small problems with operands ≥ 5 (Bagnoud et al., 2021, Poletti et al., 2023, Uittenhove et al., 2016). Because these studies have typically (i) excluded tie problems and problems involving 1 (i.e., 1-problems) and (ii) included sum-to-10 problems, we first used the same criteria and plotted RTs for the remaining problems. As can be seen on Fig. 3A (left) and B (left), we replicate in both the vocal and silent tasks the patterns of findings from previous studies (see for example Fig. 2 in Poletti et al., 2023), with an increase of RT as a function of the sum for problems with operands ≤ 4 across all groups and a plateau (or even a decrease in some groups) for problems with operands ≥ 5.

However, it has been recently argued that the plateau might be driven by sum-to-10 problems, which may have a special status in memory (Chen and Campbell, 2018). It is also not clear that 1-problems should necessarily be removed from the calculation of the slopes, as these could be theoretically computed by automatized counting. Therefore, we removed from the following behavioral and fMRI analyses sum-to-10 problems and kept 1-problems in the problem set. The patterns obtained with this set of problems can be seen on Fig. 3A (right) for the vocal task and Fig. 3B (right) for the silent task. As is clear from the figure, this selection criteria makes a PSE appear for problems with operands with operands ≥ 5 in addition to that observed for operands ≤ 4. The presence of a similar behavioral effect in both categories makes for an even more stringent test of the automatized counting theory, as the theory uniquely predicts that automatized counting should only be present for problems with operands ≤ 4. Retrieval models, in contrast, would attribute the PSE observed for problems with operands ≤ 4 and for problems with operands ≥ 5 to the same source: interference from memory (Chen and Campbell, 2018).

### 3.2 Behavioral results: quantitative assessment

Excluding ties and sum-to-10 problems while including 1-problems (see above), we then evaluated the validity of the silent production task in the scanner. We first compared the average behavioral performance associated with arithmetic problems in the vocal and silent tasks. Accuracy was estimated from all problems in the vocal task and from a small number of filler trials in which participants were cued to provide a vocal answer in the silent task (see **Methods**). Accuracy was between 90% and 100% in both tasks. Although it did not increase with age in the vocal task (F_3, 124_ = 1.20, p = 0.313, η²p = 0.28), it did increase in the silent task (F_3, 124_ = 3.46, p = 0.018, η²p = 0.08). Average RTs decreased with age in both tasks. This was the case for problems with operands ≤ 4 (vocal: F_3, 124_ = 34.8, p < 0.001, η²p = 0.46; silent: F_3, 124_ = 44.9, p < 0.001, η²p = 0.52) as well as for problems with operands ≥ 5 (vocal: F_3, 124_ = 36.5, p < 0.001, η²p = 0.47; silent: F_3, 124_ = 44.8, p < 0.001, η²p = 0.52). Across participants, average RTs in the vocal task were highly correlated with average response times in the silent task (operands ≤ 4: r = 0.825, p < 0.001; operands ≥ 5: r = 0.820, p < 0.001) (see Fig. 2B, left). More central to our current interest, we estimated the slope of the relation between RTs and problem sums (i.e., the PSE) for problems of each operand magnitude (operands ≤ 4 and operands ≥ 5). Across all participants, the slope of the PSE in the vocal task was highly correlated with the slope of the PSE in the silent task (operands ≤ 4: r = 0.653, p < 0.001; operands ≥ 5: r = 0.718, p < 0.001) (see Fig. 2B, right), suggesting that the silent task provides a valid proxy of the PSE measured using the vocal task.

We then examined the PSE in each group. Participants from the 8–9-yo group (n = 31) showed relatively steep positive PSEs for both magnitudes in the vocal task (operands ≤ 4: 157 ms, t_30_ = 4.14, p < 0.001, d = 0.74; operands ≥ 5: 122 ms, t_30_ = 6.23, p < 0.001, d = 1.12) and in the silent task (operands ≤ 4: 202 ms, t_30_ = 6.36, p < 0.001, d = 1.14; operands ≥ 5: 126 ms, t_30_ = 4.95, p < 0.001, d = 0.89) (see Fig. 4A). In line with previous studies that have often found a larger PSE for problems with operands ≤ 4 than for problems with operands ≥ 5 (Bagnoud et al., 2021, Poletti et al., 2023, Uittenhove et al., 2016), the PSE for problems with operands ≤ 4 was larger than for problems with operands ≥ 5 in the silent task (t_30_ = 2.78, p = 0.005, d = 0.50). However, there was no difference in the vocal task (t_30_ = 0.36, p = 0.362, d = 0.06). In the 8–9-yo group, we also examined whether RT increased with the size of the smallest operand. Indeed, if they use silent counting, children of that age are likely to count from the largest operand rather than the smallest (a strategy called min strategy; (Ashcraft, 1982). As expected, RT increased with the size of the smallest operand in both the vocal (operands ≤ 4: t_30_ = 4.09, p < 0.001, d = 0.74; operands ≥ 5: t_30_ = 2.98, p < 0.01, d = 0.54) and the silent task (operands ≤ 4: t_30_ = 6.08, p < 0.001, d = 1.09; operands ≥ 5: t_30_ = 2.69, p < 0.01, d = 0.48). In problems with operands ≤ 4, for example, RT increased with a unit increase in smallest operand size by 264 ms in the vocal task and 391 ms in the silent task. Because these rates are larger than the rate of silent counting (estimated in adults at around 125 ms/increment; (Landauer, 1962), this strongly suggests the use of an explicit verbal counting strategy.

As can be seen on Fig. 4B (left), the PSE was reduced in participants older than 10 (n = 97). However, it remained significantly positive in the vocal task (operands ≤ 4: 77 ms, t_96_ = 6.88, p < 0.001, d = 0.70; operands ≥ 5: 83 ms, t_96_ = 13.27, p < 0.001, d = 1.35) and in the silent task (operands ≤ 4: 69 ms, t_96_ = 6.28, p < 0.001, d = 0.64; operands ≥ 5: 54 ms, t_96_ = 8.53, p < 0.001, d = 0.87). As for participants in the 8–9-yo group, the PSE measured in the silent task was larger for problems with operands ≤ 4 than for problems with operands ≥ 5 across participants older than 10 (t_96_ = 1.96, p = 0.026, d = 0.20). However, there was no difference in the vocal task (t_96_ = −0.78, p = 0.782, d = −0.08).

Comparing all four groups (see Fig. 4B, right), the PSE significantly decreased with age in both the vocal task (operands ≤ 4: F_3, 124_ = 2.93, p = 0.036, η²p = 0.07; operands ≥ 5: F_3, 124_ = 3.26, p = 0.024, η²p = 0.07) and the silent task (operands ≤ 4: F_3, 124_ = 9.49, p < 0.001, η²p = 0.19; operands ≥ 5: F_3, 124_ = 6.68, p < 0.001, η²p = 0.14). Nevertheless, it remained significantly positive for each individual group, task, and operand magnitude (all ts > 3.09, all ps < 0.003). That is, even adult participants exhibited significant PSEs in the vocal task (operands ≤ 4: 59 ms, t_39_ = 5.03, p < 0.001, d = 0.80; operands ≥ 5: 67 ms, t_39_ = 8.56, p < 0.001, d = 1.35) and in the silent task (operands ≤ 4: 51 ms, t_39_ = 4.26, p < 0.001, d = 0.67; operands ≥ 5: 41 ms, t_39_ = 5.48, p < 0.001, d = 0.87).

### 3.3 fMRI: functional ROI definition

Functional ROIs were defined as the brain regions associated with a significant neural PSE in the 8–9-yo group. Across problems of both operand magnitudes (operands ≤ 4 and operands ≥ 5), activity significantly increased with problem sum (i.e., the neural PSE was positive) in three regions: the left IPS (x = −26, y = −62, z = 48; t-score = 4.40; cluster size = 1092 mm^3^, p_corr_ = 0.007), the medial prefrontal cortex (mPFC) (x = −12, y = 18, z = 44; t-score = 6.40; cluster size = 8736 mm^3^, p_corr_ = 0.009), and the right Insula (x = 30, y = 28, z = −1; t-score = 5.05; cluster size = 1148 mm^3^, p_corr_ = 0.027) (see Fig. 5A). There was no interaction between operand magnitude and problem sum, indicating that the slope of the neural PSE was equivalent in these regions for problems with operands ≤ 4 and problems with operands ≥ 5. Therefore, these three regions served as functional ROIs for subsequent analyses.

### 3.4 fMRI: functional ROI analyses

Average univariate activity was extracted from each functional ROI (defined in the 8–9-yo group) in the 11–12-yo, 14–15-yo, and adult groups (see Methods). Across all of these three groups, the neural PSE was positive in all three functional ROIs for problems with operands ≤ 4 (IPS: t_95_ = 4.69, p_corr_ < 0.001, d = 0.48; mPFC: t_95_ = 2.31, p_corr_ = 0.035, d = 0.24; Insula: t_95_ = 4.92, p_corr_ < 0.001, d = 0.50), but not for problems with operands ≥ 5 (IPS: t_96_ = 1.86, p_corr_ = 0.099, d = 0.19; Insula: t_96_ = 1.14, p_corr_ = 0.387, d = 0.11; Insula: t_96_ = 1.87, p_corr_ = 0.096, d = 0.19) (see Fig. 5B). To compare the neural PSE as a function of operand magnitude, ROI, and group, slopes were entered into a 2 × 3×3 ANOVA with the within-subject factors operand magnitude (operands ≤ 4, operands ≥ 5) and ROI (IPS, mPFC, Insula), and the between-subject factor group (11–12-yo, 14–15-yo, adults). There was a significant main effect of ROI (F_4, 186_ = 7.58, p < 0.001, η²p = 0.075), indicating that the neural PSE was generally steeper in the IPS and Insula than in the mPFC. More importantly, there was also a main effect of operand magnitude (F_4, 93_ = 4.13, p = 0.045, η²p = 0.042), showing that across all ROIs and across all three groups the PSE was steeper in problems with operands ≤ 4 than in problems with operands ≥ 5. In adults, for example, the neural PSE was positive for problems with operands ≤ 4 in all three ROIs (IPS: t_39_ = 4.22, p_corr_ < 0.001, d = 0.67; mPFC: t_39_ = 2.16, p_corr_ = 0.056, d = 0.34; Insula: t_39_ = 3.21, p_corr_ = 0.004, d = 0.51), but this was not the case for problems with operands ≥ 5 (IPS: t_39_ = 1.03, p_corr_ = 0.154, d = 0.16; mPFC: t_39_ = 0.93, p_corr_ = 0.178, d = 0.15; Insula: t_39_ = 0.77, p_corr_ = 0.222, d = 0.12). A direct comparison between slopes showed a steeper neural PSE for problems with operands ≤ 4 than problems with operands ≥ 5 in adults in the IPS (t_39_ = 2.30, p_corr_ = 0.040, d = 0.36), though there was no significant difference in the mPFC (t_39_ = 1.09, p_corr_ = 0.423, d = 0.17) and Insula (t_39_ = 1.90, p_corr_ = 0.099, d = 0.30).

### 3.5 fMRI: anatomical ROI analyses

The basal ganglia are known to be implicated in procedural learning (Janacsek et al., 2022) and studies suggest a role for this structure in mental arithmetic (Rivera et al., 2005, Saban et al., 2021, Zamarian et al., 2006). Therefore, average univariate activity was extracted from four anatomical ROIs of the basal ganglia (i.e., the left and right caudate and the left and right putamen) in the 8–9-yo, in the 11–12-yo, 14–15-yo, and adult groups (see Fig. 6). There was no positive neural PSE for problems with operands ≥ 5 in any group or ROI (all t_s_ < 1.21, all p_corr_ > 1, all ds < 0.22). However, we found a positive neural PSE for problems with operands ≤ 4 in the left caudate in the adult group (t_39_ = 3.34, p_corr_ = 0.029, d = 0.53). In that region, the neural PSE was steeper for problems with operands ≤ 4 than for problems with operands ≥ 5 in adults (t_39_ = 2.98, p_corr_ = 0.040, d = 0.47). Repeated-measures ANOVA further indicated that the neural PSE increased with age in the left caudate for problems with operands ≤ 4 (F_3, 124_ = 3.35, p = 0.021, η²p = 0.075), whereas this was not the case for problems with operands ≥ 5 (F_3, 124_ = 0.41, p = 0.744, η²p = 0.010).

### 3.6 fMRI: whole-brain analyses

Brain regions in which activity increased with problem sum were also investigated across the whole brain. Across all participants older than 10 (11–12-yo, 14–15-yo, and adult groups), we did not find any significant cluster showing a neural PSE for problems with operands ≥ 5. However, there were two clusters showing a positive PSE for problems with operands ≤ 4. These were the left IPS (x = −26, y = −62, z = 48; t-score = 3.62; cluster size = 1148 mm^3^, p_corr_ = 0.006) and the mPFC (x = 8, y = 16, z = 44; t-score = 6.08; cluster size = 3052 mm^3^, p_corr_ = 0.009) (see Fig. 7A). As can be seen on Fig. 7A, both clusters were largely overlapping with the IPS and mPFC clusters identified in the 8–9-yo group.

A direct comparison between participants from the 8–9-yo group and participants older than 10 did not reveal any significant difference in the size of the neural PSE across the whole brain. However, it is possible that differences in that relation might emerge more gradually over development. To investigate this possibility, we separated each age group and entered the neural PSE in a whole brain one-way ANOVA with age group as between-subject factor (8–9-yo, 11–12-yo, 14–15-yo, adults). There was no region showing a significant increase or a decrease in neural PSE for problems with operands ≥ 5. However, as shown in Fig. 7B, we identified three clusters in which the neural PSE increased with age for problems with operands ≤ 4: the left IPS (x = −34, y = −54, z = 44; t-score = 3.07; cluster size = 756 mm^3^, p_corr_ = 0.021), the dorsal precentral gyrus (dPG) (x = −40, y = −6, z = 41; t-score = 4.10; cluster size = 238 mm^3^, p_corr_ = 0.045), and the ventral precentral gyrus (vPG) (x = −38, y = 4, z = 24; t-score = 5.04; cluster size = 378 mm^3^, p_corr_ = 0.033). Increases in neural PSE over development in the left IPS were notably seen in the left IPS cluster identified in the 8–9-yo group.

## 4 Discussion

Prior support for the automatized counting model of mental arithmetic comes from the finding that a PSE is systematically observed when adding operands ≤ 4, even in adults who have long been supposed to retrieve answers from memory (Thevenot and Barrouillet, 2020). However, it has also been argued that the effect may reflect interferences from memory in adults rather and automatized counting, and that the boundary associated with operands ≤ 4 may be more apparent than real (Chen and Campbell, 2018). Here we measured changes in the neural substrates of the PSE across development to disentangle between these hypotheses.

### 4.1 The neural mechanisms associated with the PSE for addition problems with operands ≤ 4 are qualitatively similar through learning and development

Focusing on the exact same set of small problems as in prior studies (e.g., Poletti et al., 2023), we first replicated the patterns of RTs previously found, with a PSE for operands ≤ 4 and an apparent plateau with operands ≥ 5 (see Fig. 3, left). Although removing sum-to-10 problems made a PSE appear even for problems with operands ≥ 5, the PSE remained steeper for operands ≤ 4 than for operands ≥ 5, at least for the silent arithmetic task performed in the scanner. A critical question is whether that PSE reflects an accelerated counting procedure (i.e., a scanning of a sequence of numbers) that reminds of the counting procedure used by younger children or a process that would be qualitatively different in adults and children (memory retrieval versus counting).

We reasoned that the automatized counting model would predict that brain regions supporting the PSE in participants older than 10 would be qualitatively similar to the brain regions supporting the PSE in younger children (who still frequently use silent counting; Ashcraft and Fierman, 1982), but that this would be limited to problem with operands ≤ 4. Our neuroimaging results largely confirm this hypothesis. Specifically, we show that the three brain regions (mIPS, mPFC, and Insula) in which activity was associated with the PSE in children from the 8–9-yo group (when silent counting is still at least partly used) still contribute to the PSE in participants from the other age groups (who largely do not report using silent counting, Campbell and Xue, 2001). However, this is only the case for problems with operands ≤ 4. Interestingly, that limit of 4 is a unique feature of the automatized counting model. Indeed, the model assumes that with practice silent counting might turn into mental scanning of a sequence of numbers, a process that may be fast and efficient only if magnitudes of operands can be captured within a single focus of attention (Cowan, 2001, Uittenhove et al., 2016). The fact that problems with operands ≤ 4 are associated with a neural PSE in similar regions in children and adults suggest that even adults may use a process similar to counting when adding these small numbers (though the process they use may be more similar to mental scanning of a sequence of numbers than silent counting; Uittenhove et al., 2016).

### 4.2 The neural PSE for addition problems with operands ≤ 4 increases through learning and development in frontal and parietal regions, as well as in the basal ganglia

Our developmental findings also revealed a striking pattern. As demonstrated in previous studies (Bagnoud et al., 2021, Poletti et al., 2023), the PSE observed for problems with operands ≤ 4 significantly decreased with age, which likely reflects an increase in processing efficiency (either attributed to procedural acceleration or to a reduction of interferences in memory, depending on the theory; Chen and Campbell, 2018; Thevenot and Barrouillet, 2020). Yet, activity in several regions supporting the PSE in younger children did not simply stay constant in older participants, it also increased with age. This was notably the case in the IPS, as well as in the PG and basal ganglia (i.e., left caudate). Such an increase of activity associated with a decrease in behavioral cost (i.e., a reduction of the PSE with age) arguably suggests that the neural PSE observed in these regions benefits behavioral performance, rather than impedes it. This is difficult to explain with associative models, as these are more likely to consider the neural PSE as an index of interferences within a network of facts (Chen and Campbell, 2018). However, this pattern is consistent with the view that a procedure might be automatized and therefore becomes more efficient with learning. That is, for the same increase in problem sum, there is a greater increase of activity in adults than in younger children, which itself is associated with a smaller behavioral cost (the PSE being smaller in adults than children).

What might be the specific roles of the regions in which increases of activity is observed? Inferring functions from brain activity is challenging because there is no one-to-one mapping between brain regions and behavior (Poldrack, 2011). Nonetheless, we note that the developmental increase in IPS and PG activity associated with the PSE might be consistent with an automatization of spatial movement along the mental number line, which may increasingly resemble lateralized shifts of attention (Díaz-Barriga Yáñez et al., 2020, Mathieu et al., 2018a, Mathieu et al., 2018b). Indeed, a wealth of evidence indicates that the IPS supports the mental number line (Hubbard et al., 2005), and the dPG may correspond to a region of the frontal eye fields involved in shifts of attention (Mathieu et al., 2018b). Some support for this model is given by an exploratory functional connectivity analysis showing an increase in connectivity with age between the dPG and the IPS (see **Supplementary Results** and Fig. S1). Further support is given by the results in the left caudate nucleus. Given the well-established role of the basal ganglia in procedural learning (Janacsek et al., 2022), this finding is consistent with the idea that arithmetic learning involves procedural automatization in the case of problems with operands ≤ 4. More broadly, results in the basal ganglia echo with studies suggesting that subcortical structures play an important role in mental arithmetic (Saban et al., 2021, Zamarian et al., 2006).

### 4.3 Evidence for automatized counting in the previous neuroimaging literature

The present findings may appear to conflict with the often-stated claim that arithmetic learning involves a qualitative change in brain activity, with a decrease of brain activity in fronto-parietal regions subserving procedural knowledge and an increase of activity in the left temporo-parietal cortex believed to subserve memory retrieval (Cho et al., 2011, Qin et al., 2014, Rivera et al., 2005, Zamarian et al., 2009). However, most previous studies have not focused on the type of small addition problems that are investigated here. That is, what is considered *procedural knowledge* in that literature almost exclusively concerns explicit calculation procedures relying on counting, decomposition, or backup strategies (Sokolowski et al., 2022, Zamarian et al., 2009). The frequency of such effortful and conscious procedures may clearly decrease as individuals gain expertise with arithmetic, as evidenced by self-reports (Geary et al., 2012). For example, a decrease in fronto-parietal activity (paralleling a decrease in response times) is typically observed in training studies in adults (Declercq et al., 2022, Fias et al., 2021, Grabner et al., 2009, Zamarian et al., 2009). But these training studies are unlikely to promote the use of automatized counting, either because they do not focus on simple addition (Bloechle et al., 2016, Declercq et al., 2022, Grabner et al., 2009, Soltanlou et al., 2022) or teach isolated artificial arithmetic facts (Tiberghien et al., 2019). Moreover, neuroimaging studies involving self-reports say nothing about whether effortful procedures are replaced with memory retrieval or automatized procedural knowledge, as both strategies are assumed to be so fast that they are not accessible from consciousness (Chen and Campbell, 2018, Thevenot and Barrouillet, 2020).

Interestingly, some previous neuroimaging findings are suggestive of the use of automatized procedural knowledge in arithmetic. These mainly come from studies that have contrasted activity associated with different types of problems whose answers are typically considered stored in similar networks of facts by associative theories (e.g., single-digit addition, subtraction, and multiplication). For example, studies have found differences in the neural representations of these operations, both in adults and throughout development (Brunner et al., 2021, Prado et al., 2014, Prado et al., 2011). In a recent study, Tiberghien et al., 2019, found evidence that larger single-digit multiplication facts are more similar with each other than smaller single-digit multiplication facts, in line with accounts positing interferences within a network of facts. However, the same pattern was not found for single-digit addition, again suggesting that these may not rely on the same type of representation. More generally, the idea that arithmetic learning may involve procedural automatization is in keeping with a number of studies that find an increase of activity in the IPS with age (Vogel and De Smedt, 2021).

### 4.4 Lack of neural PSE associated with problems with operands ≥ 5

An interesting aspect of our results is that we only captured a neural PSE for problems with operands ≤ 4 in participants older than 10. The lack of neural PSE for problems with operands ≥ 5 may seem surprising given that the neural PSE has been one of the most consistent effects found in previous neuroimaging studies of mental arithmetic (De Smedt et al., 2011, Jost et al., 2011, Prado et al., 2011, Prado et al., 2014). However, previous studies have never investigated the PSE *within* specific categories of problems defined by different sizes of operands (e.g., ≤ 4 and ≥ 5). Rather, studies have typically investigated the PSE by focusing on brain differences *between* categories of problems that differed in average sizes (De Smedt et al., 2011, Jost et al., 2011, Prado et al., 2011, Prado et al., 2014). An issue with this approach is that differences between such categories of problems are usually confounded by relatively large differences in response times, which makes it difficult to parse out effects of time on task from effects of problem size. Here we speculate that a neural PSE is not observed within problems with operands ≥ 5 because these problems may be solved using a greater variety of strategies (e.g., including retrieval, counting, or decomposition Campbell and Xue, 2001) than problems with operands ≤ 4. This might make it more difficult to identify subtle changes in activity associated with an increase in problem sum within problems with operands ≥ 5.

### 4.5 Alternative explanations

Can our results be accounted for by alternative account? For example, one might argue that the development increase in neural PSE observed in the IPS might simply reflect increasing specialization for processing number, consistent with the interactive specialization accounts of brain functioning (e.g., Johnson, 2011). However, such a domain-general explanation is very unlikely because this account cannot explain why the development increase in neural PSE we observed is specific to problems with operands ≤ 4. If the developmental increase was reflecting a specialization for number processing, why was it not apparent for problems with operands ≥ 5?

It is also important to explore whether associative accounts may explain our findings. For instance, seminal studies have argued that only about half of 8- to 9-year-olds still clearly use counting when solving addition problems (Ashcraft and Fierman, 1982, Siegler, 1987). This leaves open the possibility that our ROIs defined in 8- to 9-year-old children might reflect a combination of explicit counting and memory retrieval. Therefore, the overlap in activity with older groups may show a stability in processes related to memory retrieval rather than counting. This is unlikely for at least four reasons. First, a share of 50% of children who would use explicit counting is arguably significant and is in stark contrast with what is observed in children older than 10, who by and large no longer report using explicit counting strategies on small addition problems (Ashcraft and Fierman, 1982). Therefore, the fact that similar regions underlie the PSE in participants younger and older than 10 remains difficult to account for memory retrieval models. Second, solving time increased with the size of the smallest operand at a rate that was larger than silent counting in 8- to 9-year-olds. This indicates the use of a min counting strategy for the majority of children in our 8–9-yo group. Third, the brain regions associated with a neural PSE in that group, involving the parietal and frontal cortex, are typically not brain regions associated with memory retrieval in the literature (Vogel and De Smedt, 2021). Fourth, we found an increase in neural PSE in these regions with age. If activity in these regions was reflecting interferences from memory, it should decrease rather than increase with age (especially for problems with operands ≤ 4, which are those with the largest neural PSE in adults). More generally, a fundamental aspect of our findings is that there was a neural dissociation in the neural PSE supporting problems with operands ≤ 4 and problems with operands ≥ 5. To our knowledge, it is unclear how associative accounts may explain this dissociation, which is only accounted for by the automatized counting model. Therefore, we believe that the automatized counting model provides the most parsimonious explanation for our results.

### 4.6 Conclusion

In sum, we found that the brain mechanisms underlying the PSE in children at the beginning of learning still underlie that PSE observed for problems with operands ≤ 4 in adults. We also found that the PSE associated with these problems is increasingly supported by both the intraparietal sulcus and the basal ganglia. These findings are difficult to account for by associative models, which typically assume arithmetic learning involves a systematic shift from procedural to memory-based processes. Rather, our findings provide support for the automatized counting model of arithmetic (Uittenhove et al., 2016).

## Declaration of Competing Interest

The authors declare that they have no known competing financial interests or personal relationships that could have appeared to influence the work reported in this paper.

## Data Availability

The link to the data is indicated in the manuscript.
