# Supplementary material for: Neural evidence for procedural automatization during cognitive development: Intraparietal response to changes in very-small addition problem-size increases with age
Source: Dev Cogn Neurosci. 2023 Oct 4;64:101310. doi: 10.1016/j.dcn.2023.101310 (PMC10570710; doi:10.1016/j.dcn.2023.101310)
Supplement: Supplementary file 1 — Supplementary material [file mmc1.docx]

**Supplementary Material**

**Neural evidence for procedural automatization during cognitive development:**

**Intraparietal response to changes in very-small addition problem-size increases with age**

Andrea Díaz-Barriga Yáñez, Léa Longo, Hanna Chesnokova, Céline Poletti, Catherine Thevenot, & Jérôme Prado

**Functional connectivity analyses**

Our main results suggest age-related increases in the neural PSE in frontal (dPG, vPG) and parietal (IPS) regions from the 8-9-yo group to the adult group. In supplementary analyses, we used PPI to assess whether connectivity between frontal regions and the IPS was specifically sensitive to problem sum, and whether this was also dependent upon operand magnitude and group. Data were also analyzed using psychophysiological interaction (PPI) analyses (Gitelman et al., 2003). A PPI analysis assesses whether certain brain areas (target regions) show activity that can be explained in terms of an interaction between the influence of a distal area (source region) and an experimental parameter. In other words, a PPI analysis tests whether activity in a source area contributes to activity in a target area to a greater (or lesser) extent in one condition versus another. Here, we aimed to assess changes in functional connectivity as a function of problem sum between a source (dPG or vPG) and different target ROIs (IPS, mPFC, Insula). The PPI model included three regressors: (i) the average time series of the seed region (i.e., the “physiological” part of the PPI), (ii) the problem sum after it had been convolved with a standard HRF (the “psychological” parts of the PPI), and (iii) the interaction between the physiological and psychological factors (i.e., the “interaction” part of the PPI). To compute this interaction regressor, the BOLD signal from the seed region was deconvolved by using a Bayesian estimation algorithm (Gitelman et al., 2003). The regressor coding for problem sum was then multiplied to the deconvolved seed activity regressor to produce the interaction term. This interaction term was then convolved with a standard HRF. This interaction term was estimated separately for each target ROI, group and operand magnitude (problems with operands ≤ 4 and problems with operands ≥ 5). It quantified the degree to which activity of a given target ROI was explained by the interaction between activity of the source region and an increase in problem sum. Parameter estimates were then submitted to second-level one-sample t-tests across participants to identify target ROIs for which increases in connectivity with the source area as a function of the problem sum was greater than 0. One-tailed p values less than 0.05, corrected for multiple comparisons across all ROIs using the Bonferroni method, were considered to be significant.

Using either the dPG or the vPG as a seed region and the three ROIs as target regions (IPS, mPFC, and Insula), sum-related increases in connectivity were entered in a 2 x 2 x 3 ANOVA with the within-subject factors operand magnitude (operands ≤ 4, operands ≥ 5) and ROIs (IPS, mPFC, and Insula) and the between-subject factor group (8-9-yo, 11-12-yo, 14-15-yo, adults). Although there was no significant main effect or interaction with the vPG as seed (all Fs < 2.78, all ps > 0.064), there was a significant interaction between operand magnitude, ROI and group with the dPG as seed (F_6, 248_ = 2.32, p = 0.034, η²p = 0.012). As can be seen on **Fig. S1**, sum-related increases in connectivity did not vary with group in any target ROI for operands ≥ 5. However, it increased with age group for operands ≤ 4, but only in the IPS. Specifically, sum-related increases in connectivity between the dPG and IPS were not significant in the 8-9-yo group (t_30_ = -0.09, p_corr_ > 0.999, d = -0.017), 11-12-yo group (t_30_ = 0.39, p_corr_ > 0.999, d = 0.0702), or 14-15-yo group (t_25_ = 1.44, p_corr_ = 0.240, d = 0.283). But they were significant in the adult group (t_39_ = 2.35, p_corr_ = 0.036, d = 0.372). In the adult group, sum-related increases in connectivity between the dPG and IPS were also larger for problem with operands ≤ 4 than for problems with operands ≥ 5 (t_39_ = 2.63, p_corr_ = 0.012, d = 0.416). Therefore, sum-related increases of connectivity between the dPG and the IPS became larger with age, suggesting an increase in functional coupling between the dPG and IPS when the problem sum increased.


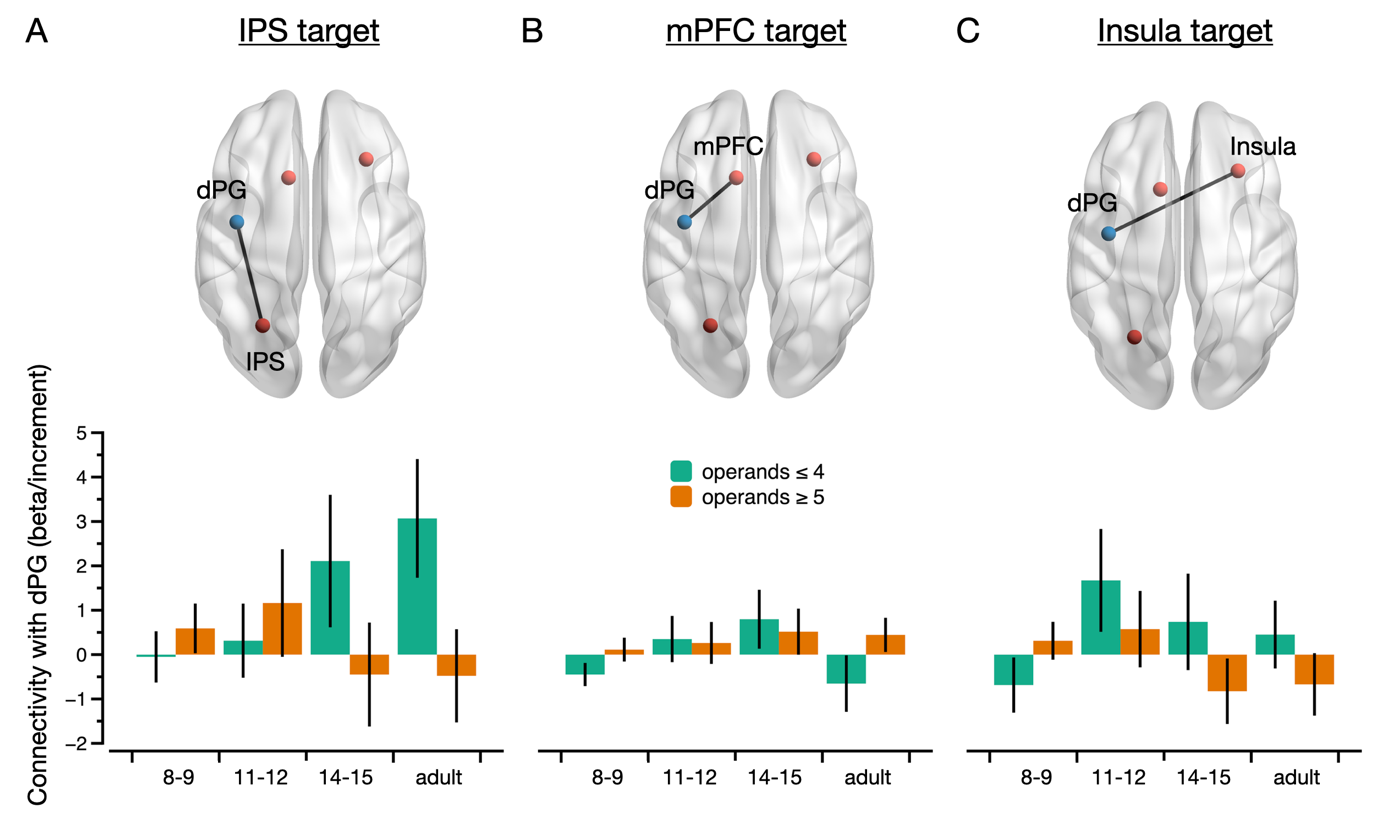


**Figure S1.** Functional connectivity between the dPG (seed) and the ROIs (targets). (A) Sum-related increases in connectivity between the dPG and the IPS as a function of age group and magnitude of operands. (B) Sum-related increases in connectivity between the dPG and the mPFC as a function of age group and magnitude of operands. (C) Sum-related increases in connectivity between the dPG and the Insula as a function of age group and magnitude of operands. Error bars represent standard error of the mean.

**References**

Gitelman, D.R., Penny, W.D., Ashburner, J., Friston, K.J., 2003. Modeling regional and psychophysiologic interactions in fMRI: the importance of hemodynamic deconvolution. Neuroimage 19, 200–207. <https://doi.org/10.1016/s1053-8119(03)00058-2>
